# Nanomaterial-enabled physical rehabilitation: mechanisms, evidence, and translational pathways

**DOI:** 10.3389/fresc.2026.1763655

**Published:** 2026-04-13

**Authors:** Sarfaraz K. Niazi

**Affiliations:** College of Pharmacy, University of Illinois, Chicago, IL, United States

**Keywords:** magnetic nanoparticles, nanomaterials, neuromodulation, photothermal therapy, physical rehabilitation, sonodynamic therapy, tissue regeneration, translational nanomedicine

## Abstract

Physical rehabilitation relies on macroscopic therapeutic modalities such as ultrasound, photothermal stimulation, electrical activation, magnetic fields, and controlled mechanical loading to restore function after injury, surgery, or neurological impairment. Although these approaches are clinically established and widely used, their effects remain constrained by limited spatial precision, shallow penetration, heterogeneous tissue responses, and insufficient control over the cellular and molecular mechanisms that govern healing. Advances in nanoscience have introduced a new class of materials capable of mediating, amplifying, or refining external physical stimuli at the nanoscale. Nanomaterials exhibit tunable optical, magnetic, mechanical, and electrical properties that enable the conversion of externally applied energy into localized thermal, mechanical, or electrochemical cues, thereby influencing cellular behavior with a degree of precision not achievable by conventional modalities alone. These properties suggest potential—demonstrated primarily in preclinical models—to improve musculoskeletal repair, modulate nociceptive pathways, enhance neuromuscular activation, and integrate with regenerative scaffolds, though clinical validation remains limited. Yet, despite promising experimental findings, translation into rehabilitation practice remains limited by gaps in mechanistic understanding, variability in experimental design, safety uncertainties, and complex regulatory pathways. This review examines the fundamental properties of nanomaterials relevant to physical rehabilitation, analyzes their interactions with primary therapeutic modalities, evaluates preclinical and early clinical evidence, and outlines translational, safety, and regulatory considerations. By synthesizing mechanistic insight with empirical data, the review defines realistic opportunities and the limitations that must be resolved to advance nanomaterial-enabled physical rehabilitation toward clinical implementation.

## 1 Introduction

Physical rehabilitation constitutes a foundational domain of clinical medicine, essential for restoring function in musculoskeletal, neurological, and chronic pain conditions (1, 2). Modalities such as therapeutic ultrasound, photothermal and photobiomodulation therapies, transcutaneous and functional electrical stimulation, magnetic-field-based interventions, and structured mechanotherapy have demonstrated benefits in pain reduction, tissue repair, neuromuscular activation, and functional recovery (3–5). Despite decades of clinical use, their efficacy remains limited by the macroscopic nature of energy delivery and the complex heterogeneity of biological tissues. Ultrasound produces spatially variable heating and mechanical stresses influenced by tissue anisotropy and cavitation thresholds (6, 7). Light-based modalities exhibit wavelength-dependent scattering and absorption, which limit penetration depth and confine therapeutic action to superficial tissues (8, 9). Electrical stimulation must overcome high impedance and often lacks selectivity for targeted neural structures (1, 10). Mechanical loading elicits beneficial adaptive responses but cannot directly manipulate molecular pathways governing regeneration (4, 11).

In contrast, advances in nanoscience reveal that engineered nanomaterials possess unique physicochemical properties arising from their high surface-to-volume ratios, tunable surface chemistries, and size-dependent optical, magnetic, electrical, and mechanical characteristics (12, 13). Their ability to interact intimately with cellular and extracellular structures has enabled breakthroughs in drug delivery, imaging, and regenerative medicine (14–16). Metallic nanoparticles, metal oxide nanostructures, carbon-based nanomaterials, polymeric carriers, and electrospun nanofiber scaffolds each offer distinct modes of energy absorption and transduction that make them suited for integration with external therapeutic stimuli (17–21). This convergence has produced a compelling conceptual shift: instead of delivering physical stimuli broadly and hoping for desired biological effects, nanomaterials can act as nanoscale mediators that localize, amplify, or modulate energy in ways conventional modalities cannot achieve.

Photothermal nanostructures convert near-infrared light into precise heat doses that can modulate nociception or stimulate tissue remodeling (22, 23). Superparamagnetic iron oxide nanoparticles respond to alternating magnetic fields by producing controlled hyperthermia or mechanical forces that can modulate deep tissues without attenuation (24, 25). Carbon nanotubes, graphene, and conductive polymers improve neural-electrode interfaces by reducing impedance and increasing charge-injection capacity (10, 26, 27). Nanofiber scaffolds can recreate extracellular matrix topographies that guide cell alignment, differentiation, and mechanotransduction (11, 28). Yet the integration of nanotechnology into physical rehabilitation is still in its early stages. Much of the evidence supporting nano-enabled modalities derives from oncology, imaging, or regenerative medicine research rather than rehabilitation-specific trials. Differences in therapeutic goals—such as reversible neuromodulation, sublethal photothermal conditioning, functional tissue repair, or controlled mechanical reinforcement—require distinct mechanistic considerations (5, 29, 30). Experimental variability in nanomaterial formulations, dosing, and energy parameters further complicates interpretation (7, 31). Some proposed mechanisms remain speculative and lack rehabilitation-specific validation; these are discussed briefly in Section 5.4 as potential future directions rather than current therapeutic options.

Safety and regulatory concerns are particularly salient. Nanomaterials can accumulate in the liver, spleen, and lymphoid organs, activate complement pathways, or interfere with normal immune function if their physicochemical properties are not carefully controlled (32–35). Regulatory agencies have issued guidance emphasizing rigorous characterization, reproducible manufacturing, and long-term biodistribution studies for products incorporating nanomaterials (36, 37). These requirements apply equally—and in some cases more stringently—to nano-enabled physical therapies that combine both device and drug characteristics. This review, therefore, aims to provide a comprehensive and critical synthesis of nanomaterial-enabled physical rehabilitation, integrating mechanistic principles with empirical findings and translational considerations. The analysis examines the fundamental design parameters that determine nanomaterial behavior in biological environments, the mechanisms by which nanomaterials interact with primary rehabilitation modalities, preclinical and early clinical evidence supporting these combined approaches, and the safety, regulatory, and ethical dimensions necessary for clinical translation. By clarifying both opportunities and limitations, the review seeks to establish a realistic scientific foundation for advancing nanomaterial-enabled rehabilitation toward clinical practice.

## 2 Nanomaterial classes and design principles for therapeutic applications

The therapeutic potential of nanomaterials in physical rehabilitation arises from the interplay between their physicochemical properties and their interactions with biological systems. These properties include composition, dimensionality, size distribution, shape anisotropy, surface chemistry, and energy transduction behavior. Each of these attributes influences biodistribution, cellular uptake, clearance, immune recognition, and the capacity to modulate local biological responses under external physical stimuli. A mechanistic understanding of these parameters is essential for designing nanomaterials that can improve the precision and efficacy of physical therapy modalities.

### 2.1 Dimensional classification of therapeutic nanomaterials

Nanomaterials used in biomedical applications are commonly categorized according to dimensionality—zero-dimensional nanoparticles, one-dimensional nanofibers or nanotubes, two-dimensional nanosheets, and three-dimensional nanocomposite hydrogels. This classification reflects differences in surface area, aspect ratio, and directional constraints, all of which influence therapeutic interactions.

#### 2.1.1 Zero-dimensional nanoparticles

Zero-dimensional nanoparticles, typically 1–100 nm in size, represent the most widely studied class of therapeutic nanomaterials. Metallic nanoparticles—particularly gold and silver—demonstrate unique optical properties arising from localized surface plasmon resonance, enabling strong absorption and scattering in the visible-to-near-infrared range (22, 23). Gold nanorods, nanoshells, nanocages, and nanostars can be engineered to have resonance peaks that match the near-infrared optical window optimal for biological tissues, enabling highly efficient photothermal conversion. Silver nanoparticles, while valued for antimicrobial activity, require careful dose control due to concentration-dependent cytotoxicity (33, 38). Metal oxide nanoparticles constitute another major subclass. Superparamagnetic iron oxide nanoparticles (SPIONs) are widely used because they exhibit strong magnetization under external magnetic fields yet no remanence after field removal, preventing aggregation (17, 18). Their capacity to generate heat through Néel and Brownian relaxation mechanisms in alternating magnetic fields enables controlled magnetothermal therapy (24). Titanium dioxide nanoparticles possess photocatalytic properties and can act as sonosensitizers under ultrasound exposure, producing reactive oxygen species useful for tissue modulation or antimicrobial applications (39, 40).

Polymeric nanoparticles made of PLGA, PCL, chitosan, and PEG-based copolymers offer biocompatibility and biodegradability, enabling sustained drug release over hours to months (15, 41). Liposomes and polymersomes enable encapsulation of hydrophilic and hydrophobic agents, and thermosensitive liposomes release drugs under mild hyperthermia, making them attractive for combination therapies with ultrasound or photothermal modalities (14, 42). Quantum dots—semiconductor nanocrystals with size-tunable fluorescence—offer distinct imaging properties but raise concerns about their toxicity due to heavy-metal cores, driving the development of carbon- and silicon-based alternatives (33, 43).

#### 2.1.2 One-Dimensional nanomaterials

One-dimensional nanomaterials, such as electrospun nanofibers and carbon nanotubes, provide high aspect ratios that influence both mechanical and electrical behavior. Electrospun nanofibers with diameters of 100–1,000 nm mimic the fibrillar architecture of the extracellular matrix and can direct cell alignment, differentiation, and migration in musculoskeletal tissues (19, 21). They are commonly fabricated from polymers such as PCL, PLGA, collagen, and silk fibroin, each offering tunable mechanical strength and degradation kinetics (44, 45). Carbon nanotubes (CNTs) exhibit exceptional mechanical strength, high Young's modulus, and electrical conductivity exceeding 10⁶ S/m (45). Their ability to support charge transfer makes them valuable in electrotherapy and neural interface applications, although concerns regarding biopersistence and potential fiber-like pathology necessitate careful surface modification and dose control (20, 34).

#### 2.1.3 Two-Dimensional nanomaterials

Two-dimensional nanomaterials, particularly graphene and graphene oxide, provide extremely high surface areas and unique optoelectronic properties. Graphene oxide, with its oxygen-containing functional groups, is readily dispersed in aqueous solution and can be functionalized via carbodiimide chemistry, making it suitable for drug loading and the formation of conductive composites (46). Transition metal dichalcogenides, such as MoS₂, provide photothermal and photodynamic activity with potential advantages in biodegradability (47). Two-dimensional black phosphorus (BP) nanosheets have emerged as particularly promising materials for rehabilitation applications due to their layer-dependent bandgap, high carrier mobility, and inherent biodegradability. Unlike graphene, BP exhibits a direct bandgap tunable from 0.3 to 2.0 eV depending on layer thickness, enabling broad-spectrum light absorption suitable for photothermal applications across the near-infrared range. BP nanosheets demonstrate efficient photothermal conversion with absorption coefficients comparable to those of gold nanostructures, and their natural degradation to biocompatible phosphate ions addresses long-term accumulation concerns that limit the use of metallic nanoparticles. Recent studies have explored BP-based hydrogels for bone regeneration and neural tissue engineering, demonstrating enhanced osteogenic differentiation and improved nerve regeneration under near-infrared stimulation (48, 49). BP's biodegradability profile—complete degradation within weeks under physiological conditions—makes it particularly suited for rehabilitation applications requiring temporary scaffold support followed by complete clearance.

#### 2.1.4 Three-Dimensional nanostructured hydrogels and composites

Three-dimensional nanostructured hydrogels incorporate nanoparticles or nanofibers into hydrated polymer matrices to replicate tissue-like mechanical properties. Hydrogels made from alginate, PEG, and PNIPAM can achieve elastic moduli that match those of soft tissues and function as injectable carriers for drugs, cells, or nanoparticles (16, 50). Nanoparticle–hydrogel composites enable stimulus-responsive behavior, such as photothermal or magnetothermal triggering of drug release (51, 52). Incorporation of hydroxyapatite nanoparticles enhances osteoconductivity in bone regeneration models (44), while CNT reinforcement improves mechanical strength and electrical conductivity for nerve or muscle engineering (53). Recent advances have expanded the fabrication toolkit for therapeutic hydrogels. PEDOT: PSS conductive hydrogels are typically prepared via electrochemical polymerization, enabling precise control over film thickness and conductivity (54). GelMA (gelatin methacrylate) and PEGDA (poly(ethylene glycol) diacrylate) hydrogels are fabricated using photolithographic patterning, where UV crosslinking through photomasks creates defined microarchitectures that guide cell organization. Nanogels and microgels are increasingly produced via microfluidic platforms that generate monodisperse particles with tunable size distributions. Physical crosslinking through temperature-induced gelation (PNIPAM) or ionic crosslinking (alginate with Ca²+) offers advantages for injectable formulations that gel *in situ*. Smart hydrogels incorporating multiple responsive elements—temperature, pH, light, magnetic fields, and mechanical loading—enable programmable drug release and adaptive mechanical properties (Advanced Functional Materials, e13988, 2025). Scalability and long-term stability remain challenges for clinical translation. Manufacturing reproducibility requires stringent control of crosslinking density, nanoparticle distribution, and polymer molecular weight. Batch-to-batch variability can significantly affect mechanical properties and drug release kinetics. Long-term mechanical stability under cyclic loading—critical for rehabilitation applications involving repeated joint motion—requires fatigue testing protocols not yet standardized. Sterilization compatibility presents additional constraints, as gamma irradiation can degrade polymers while ethylene oxide may leave cytotoxic residues.

### 2.2 Critical design parameters for therapeutic nanomaterials

The design of therapeutic nanomaterials requires careful optimization of multiple physicochemical parameters that collectively determine biological behavior and therapeutic efficacy. Table 1 summarizes the key design parameters and their significance for physical rehabilitation applications.

**Table 1:** Physicochemical design parameters of therapeutic nanomaterials relevant to physical rehabilitation.

Design Parameter	Therapeutic Significance	Representative Evidence
Size (nm)	Determines biodistribution, renal versus RES clearance, and ability to penetrate fibrotic or inflamed tissue	(55–57)
Surface charge (*ζ*-potential)	Governs protein corona formation, opsonization, and complement activation	(58, 59)
Shape (spherical, rod-like, star-shaped)	Strong influence on optical absorption, photothermal efficiency, and macrophage uptake	(60, 61)
Material composition (Au, SPIONs, CNTs, PLGA, hydrogels)	Determines modality-specific responsiveness (light, magnetic field, ultrasound, mechanical load)	(17, 19, 22)
Surface functionalization (PEG, peptides, antibodies)	Enables targeting, reduces immunogenicity, and improves circulation times	(14, 62, 63)

#### 2.2.1 Size and biodistribution

Nanoparticle size strongly influences biodistribution and clearance. Particles <5–6 nm are typically rapidly filtered by the kidney, yielding short plasma half-lives (55). Particles between 10 and 100 nm achieve longer circulation times and improved tissue penetration, particularly in inflamed or damaged tissues with enhanced permeability (56). Larger particles (>200 nm) are more likely to be sequestered by the reticuloendothelial system, limiting bioavailability unless surface chemistry is optimized (57). Shape also affects pharmacokinetics and cellular uptake. Rod-shaped nanoparticles exhibit prolonged circulation and enhanced photothermal conversion efficiency due to electromagnetic field concentration at their tips (60). Worm-like or discoidal geometries may evade macrophage clearance more effectively than spherical particles (61).

#### 2.2.2 Surface chemistry, protein Corona, and immune recognition

Nanoparticle surfaces govern interactions with blood proteins and immune cells through the formation of a dynamic protein corona, which determines biological identity (59). PEGylation reduces protein adsorption but may induce anti-PEG antibodies upon repeated administration, accelerating clearance (62, 64). Zwitterionic coatings such as phosphorylcholine reduce opsonization without inducing immunogenicity (63). Surface charge influences cell uptake, with positively charged particles exhibiting enhanced interaction with negatively charged cell membranes but also increased cytotoxicity and hemolysis risk (58). Ligand conjugation to peptides, antibodies, aptamers, or small molecules enables targeted delivery to specific cell types or tissues, thereby improving local accumulation while reducing systemic exposure (14, 65).

#### 2.2.3 Energy transduction mechanisms

Nanomaterials' ability to convert external energy into localized biological effects distinguishes them from conventional therapeutic agents. Photothermal conversion in gold nanostructures relies on plasmonic resonance, with efficiencies exceeding 90% for optimized geometries (66). Photodynamic mechanisms require encapsulated photosensitizers capable of generating singlet oxygen or free radicals under light exposure, with nanocarriers improving solubility and protecting against photobleaching (67, 68). Ultrasound-responsive nanomaterials generate cavitation or acoustic vaporization, enhancing mechanical disruption and drug release (69). Magnetic nanoparticles generate heat under alternating magnetic fields or mechanical forces under low-frequency rotation, enabling deep-tissue hyperthermia or mechanostimulation (24, 70). Carbon-based materials and conductive polymers facilitate charge transfer, reducing stimulation thresholds in electrotherapy (10, 26). Nanofiber scaffolds transduce mechanical loading into biochemical signaling, activating mechanotransduction pathways such as YAP/TAZ or focal adhesion kinase (11, 28).

## 3 Mechanistic integration of nanomaterials with physical therapy modalities

**Figure 1:**
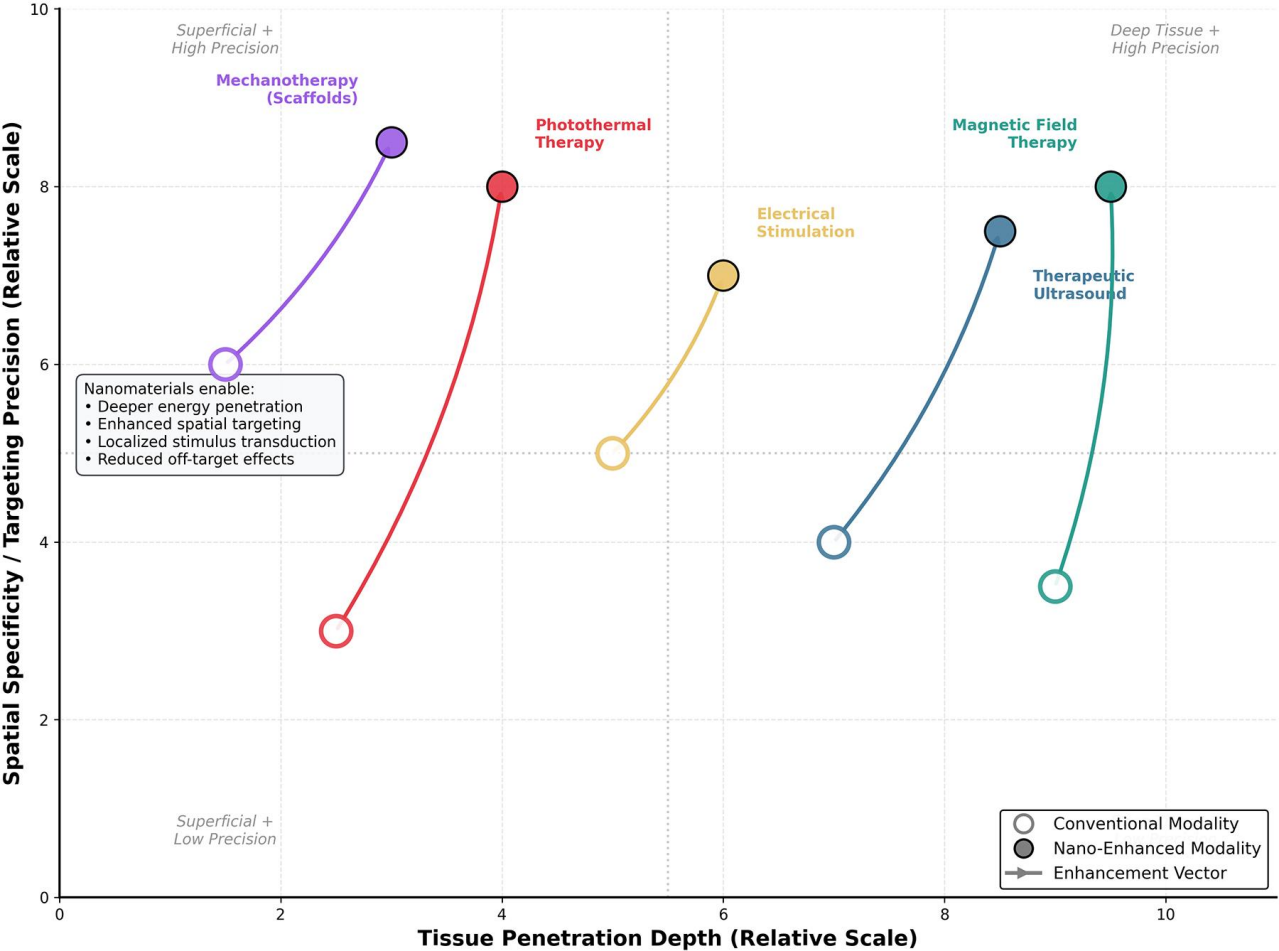
Mechanisms of nanoscale energy transduction under major rehabilitation stimuli.

Nanomaterial-enabled physical rehabilitation is founded on the ability of nanoscale systems to interact with externally applied physical stimuli, thereby amplifying, localizing, or refining therapeutic effects. Unlike conventional physical modalities, which deposit energy broadly, nanomaterials concentrated within tissues can transduce applied stimuli with nanoscale resolution, thereby influencing cellular pathways central to pain modulation, tissue repair, and functional recovery. The mechanisms by which nanomaterials convert light, ultrasound, magnetic fields, electrical currents, and mechanical forces into biological signals are diverse, stimulus-specific, and strongly dependent on material design parameters described in the previous section. Figure 1 depicts the five principal mechanisms by which nanomaterials transduce externally applied physical stimuli into localized biological effects. Panel A illustrates photothermal conversion, in which plasmonic nanostructures absorb near-infrared light and dissipate energy as localized heat via nonradiative decay. Panel B shows sonodynamic activation, in which ultrasound induces cavitation-enhanced generation of reactive oxygen species (ROS) from sonosensitizer-loaded carriers. Panel C demonstrates magnetothermal heating through Néel and Brownian relaxation in superparamagnetic iron oxide nanoparticles under alternating magnetic fields. Panel D depicts charge transfer at nano-enhanced electrode interfaces, where carbon nanotubes and conductive polymers reduce impedance and improve neural coupling. Panel E illustrates mechanotransduction through nanofiber scaffold deformation, activating cellular signaling pathways including YAP/TAZ and focal adhesion kinase.

**Figure 2:**
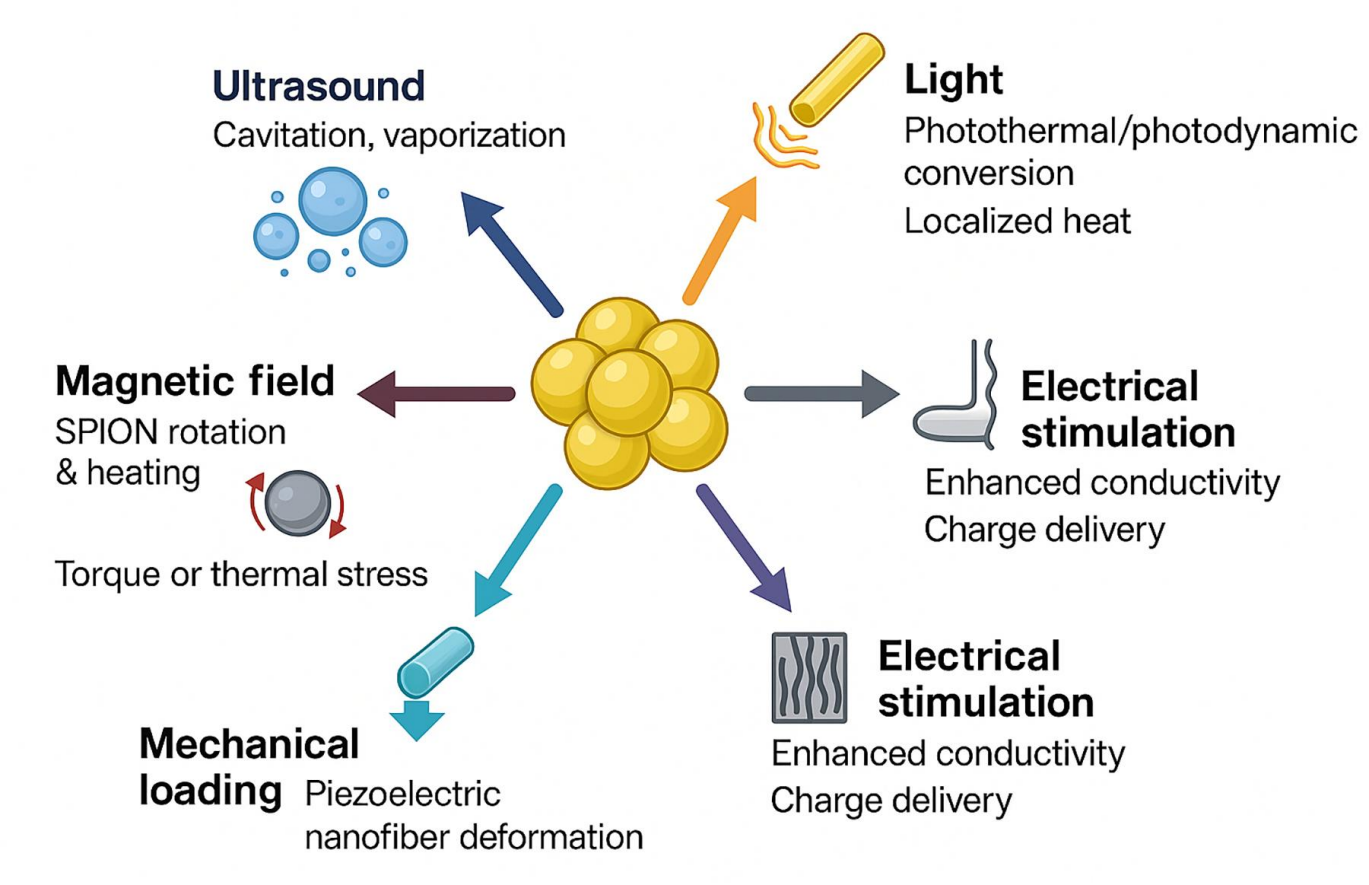
Penetration depth versus spatial specificity of physical modalities.

**Figure 3:**
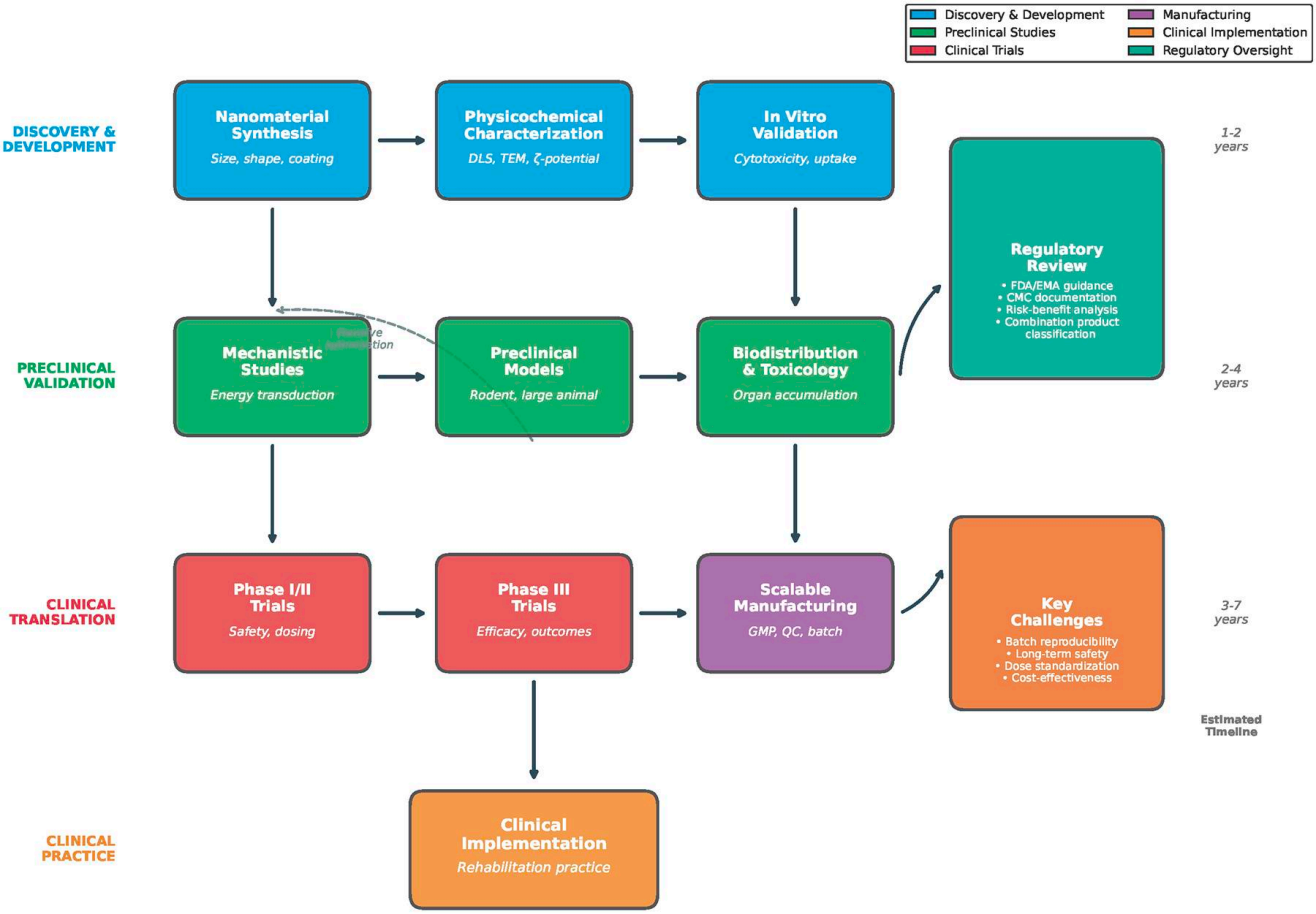
Translational pathway for nano-enabled rehabilitation from bench to bedside.

### 3.1 Nanomaterial–light interactions: photothermal and photodynamic mechanisms

Light-based therapies, including low-level laser therapy, photobiomodulation, and controlled photothermal interventions, have a long clinical history but are limited by wavelength-dependent scattering, absorption, and shallow penetration depths in biological tissues (8, 9) (Figures 2, 3). Nanomaterials enable more precise and efficient use of light by serving as absorbers or photochemical catalysts that convert incident photons into heat or reactive oxygen species with high spatial specificity.

#### 3.1.1 Photothermal conversion by plasmonic nanostructures

Gold nanostructures—especially nanorods, nanoshells, nanocages, and nanostars—exhibit localized surface plasmon resonance, wherein conduction electrons oscillate coherently under illumination at specific wavelengths (22, 23). When tuned to absorb near-infrared light, which penetrates tissue more effectively, these nanostructures convert photon energy into heat via nonradiative decay pathways. Conversion efficiencies can exceed 90%, enabling localized heating within the therapeutic range of 41–45°C, which is sufficient to modulate pain fibers, remodel extracellular matrix, and activate heat shock protein pathways involved in tissue regeneration (31, 71). The 41–45°C therapeutic range aligns with established physical therapy guidelines for deep tissue heating: temperatures above 40°C increase collagen extensibility, while those exceeding 45°C risk protein denaturation and tissue damage. Typical rehabilitation sessions employing conventional diathermy maintain tissue temperatures of 40–43°C for 15–30 min (1). Nanomaterial-mediated heating offers the advantage of more precise spatial confinement, potentially enabling shorter exposure times while achieving equivalent therapeutic heating at the target site. Session durations in preclinical studies typically range from 5 to 15 min with NIR power densities of 0.5–2.0 W/cm², though optimal parameters for specific rehabilitation applications remain to be standardized. Photothermal confinement is essential for rehabilitation applications, which often require sublethal heating rather than ablation. By selectively accumulating nanomaterials within injured, inflamed, or fibrotic tissues, photothermal effects can be focused on regions of pathological stiffness or nociceptive sensitivity, improving therapeutic precision relative to surface laser applications alone.

#### 3.1.2 Nanomaterial-Assisted photodynamic therapy

Photodynamic therapy traditionally involves small-molecule photosensitizers that generate singlet oxygen and other reactive oxygen species (ROS) upon light activation (67). Nanocarriers improve photosensitizer solubility, reduce aggregation-induced quenching, and enable targeted accumulation within tissues through ligand conjugation or passive retention. ROS have extremely short diffusion distances and lifetimes, allowing nanomaterial-enabled photodynamic therapy to exert spatially confined oxidative effects that can facilitate wound healing, reduce microbial burden, or modulate inflammation (68, 72). In musculoskeletal tissues, controlled ROS generation has been shown to promote matrix remodeling and stimulate angiogenesis when administered at subcytotoxic doses, although dosing precision is essential to avoid impaired healing (72).

#### 3.1.3 Combined photothermal–photodynamic platforms

Hybrid nanostructures that incorporate both plasmonic metals and photosensitizers enable synergistic photothermal-photodynamic effects. Mild photothermal heating increases tissue oxygenation, overcoming a common limitation of photodynamic therapy in hypoxic tissue environments (73). Conversely, ROS generation can sensitize cells to thermal stress, enabling therapeutic effects at lower temperatures. Dual-mode platforms have demonstrated improved modulation of pain pathways, fibrotic remodeling, and wound healing in preclinical studies, though careful dose control remains critical to avoid unwanted damage.

### 3.2 Nanomaterial–ultrasound interactions

Ultrasound-based therapies rely on mechanical wave propagation, which induces thermal effects, acoustic streaming, and cavitation (3, 6). Nanomaterials enhance these effects through mechanisms that reduce cavitation thresholds, increase local acoustic absorption, or act as reservoirs for ultrasound-triggered drug release.

#### 3.2.1 Cavitation enhancement by microbubbles and nanobubbles

Microbubbles, typically 1–10 μm in diameter, undergo volumetric oscillation under low-intensity ultrasound, generating shear stress and transient membrane permeabilization that improve drug uptake and tissue perfusion (7, 74). Nanobubbles (<1 μm) circulate longer and penetrate deeper tissues, though they generate weaker cavitation forces (69). In rehabilitation contexts, cavitation-enhanced ultrasound has shown potential to improve the permeability of tendons, muscles, and cartilage to therapeutics while minimizing thermal damage.

#### 3.2.2 Acoustic droplet vaporization in phase-change nanodroplets

Perfluorocarbon nanodroplets remain liquid at physiological temperatures but vaporize into gas bubbles when exposed to ultrasound, expanding several-fold in volume—a process termed acoustic droplet vaporization (ADV) (69). ADV generates localized mechanical disruption, enhances cavitation, and triggers the release of encapsulated drugs or growth factors. Studies have demonstrated accelerated tendon and cartilage healing when combining nanodroplets with ultrasound-triggered release of regenerative agents (75, 76).

#### 3.2.3 Sonodynamic therapy

Sonodynamic therapy parallels photodynamic therapy but uses ultrasound to activate sensitizers such as titanium dioxide, porphyrins, or organic dyes (40, 77). Cavitation collapse produces extreme localized temperatures and pressures, generating ROS through sonoluminescence or mechanochemical activation. In rehabilitation, sonodynamic therapy is being explored to reduce chronic inflammation, modulate fibroblast behavior, and address neuromuscular pathologies through controlled oxidative signaling.

### 3.3 Nanomaterial–magnetic field interactions

Magnetic-field-based therapies benefit from the deep penetration and minimal attenuation of low-frequency magnetic fields in biological tissues (78). When combined with magnetic nanoparticles, these fields can induce thermal, mechanical, or transport phenomena relevant to physical rehabilitation.

#### 3.3.1 Magnetothermal heating by superparamagnetic nanoparticles

SPIONs generate heat when subjected to alternating magnetic fields through Néel and Brownian relaxation (24). Unlike optical or ultrasound modalities, magnetic hyperthermia can reach deep tissues, such as the hip, spine, and deep muscle groups, without attenuation. In rehabilitation models, magnetothermal heating has been used to modulate nociceptive fibers, reduce synovial inflammation, and promote tissue remodeling (25, 79).

#### 3.3.2 Magneto-mechanical stimulation

Rotating or oscillating magnetic fields exert torque on SPIONs, producing mechanical forces that can activate mechanosensitive ion channels in neurons, stem cells, or musculoskeletal tissues (70). Magneto-mechanical stimulation has been shown to promote chondrogenic differentiation, enhance tendon matrix deposition, and facilitate nerve regeneration in preclinical models (79, 80).

### 3.4 Nanomaterial–electrical interactions

Electrical stimulation is central to many rehabilitation protocols, particularly in restoring neuromuscular function. Nanomaterials can improve electrical interfaces by reducing impedance, enhancing charge injection, and providing more intimate coupling with target cells.

#### 3.4.1 Conductive nanomaterials at electrode interfaces

Carbon nanotube coatings reduce electrode impedance by several orders of magnitude and improve signal fidelity in neural recordings and stimulation (26, 27). PEDOT:PSS and other conductive polymers form soft, conformal coatings that increase charge capacity while minimizing tissue irritation (10). These enhancements allow stimulation at lower voltages, reducing discomfort and risk of tissue damage.

#### 3.4.2 Injectable conductive scaffolds

Graphene oxide and CNT-based hydrogels can serve as injectable conductive media that bridge neural gaps or enhance muscle activation during electrical stimulation (46). These scaffolds reduce the voltage needed for muscle activation and may synergize with rehabilitative electrical protocols. Injectable conductive hydrogels incorporating graphene-based materials have shown promise for improving neuromuscular responses to therapeutic electrical stimulation (81).

### 3.5 Nanomaterial–mechanical interactions

Mechanical loading drives tissue adaptation through mechanotransduction pathways that convert physical forces into biochemical signals. Nanomaterials can modulate or enhance these pathways through several mechanisms.

#### 3.5.1 Nanofiber scaffolds and mechanotransduction

Electrospun nanofibers reproduce the alignment, stiffness, and topography of native extracellular matrix, guiding tenocytes, chondrocytes, myoblasts, and neural cells toward functional phenotypes (19, 28). Mechanical loading of nanofiber scaffolds activates YAP/TAZ, focal adhesion kinase, and cytoskeletal remodeling pathways that regulate differentiation and matrix production (4, 11).

#### 3.5.2 Piezoelectric nanomaterials

Piezoelectric materials such as PVDF nanofibers generate localized electric potentials when stretched or compressed, enabling self-powered electrostimulation during movement or therapeutic loading (82). These effects have shown promise in cartilage, tendon, and nerve regeneration.

## 4 Modality-Specific evidence for nanomaterial-enabled physical rehabilitation

The translation of nanomaterial-enabled interactions into therapeutic benefit depends on empirical evidence demonstrating biological efficacy, safety, and functional improvement in relevant models. While much of the literature remains preclinical, and clinical adoption remains limited, existing data provide meaningful insights into how nanomaterials can enhance musculoskeletal repair, modulate pain pathways, accelerate wound healing, and support neuromuscular rehabilitation. Table 2 summarizes representative preclinical and early clinical outcomes across the principal modalities.

**Table 2:** Representative preclinical and early clinical outcomes of nano-enabled physical modalities.

Modality	Nanomaterial Type	Outcome	Representative Evidence
Photothermal therapy	Gold nanorods, nanoshells	Reduced pain, reduced fibrosis, enhanced remodeling	(31, 83)
Sonodynamic/ultrasound-enhanced therapy	Nanobubbles, TiO₂ sensitizers, nanodroplets	Accelerated fracture healing; enhanced tendon/collagen repair	(40, 76, 84)
Magnetothermal therapy	SPIONs	Reduced synovial inflammation; enhanced MSC chondrogenesis	(25, 79)
Electrotherapy enhancement	CNTs, PEDOT:PSS, graphene hydrogels	Lower stimulation voltages; improved neuromuscular activation	(26, 27)
Mechanotherapy (scaffolds)	Aligned nanofibers, piezoelectric nanomaterials	Enhanced tendon alignment, cartilage regeneration, and muscle recovery	(28, 82, 85)

### 4.1 Photothermal and photodynamic nanotherapies in rehabilitation

#### 4.1.1 Musculoskeletal pain modulation

Photothermal heating in the mild therapeutic range (41–45°C) has been shown to selectively modulate nociceptive pathways. Gold nanorods that accumulate within inflamed synovial or periarticular tissues can convert near-infrared light into localized heat, producing reductions in chronic pain behaviors through thermal modulation of unmyelinated C-fibers, which demonstrate greater sensitivity to sublethal hyperthermia compared with myelinated fibers responsible for motor function (31, 71). In rodent models of arthritis, gold nanoparticle treatment has demonstrated anti-inflammatory effects by suppressing NF-*κ*B signaling and COX-2 activity, thereby reducing inflammatory cytokines and joint inflammation (83). Beyond molecular markers, preclinical studies have assessed functional rehabilitation endpoints. Gold nanoparticle-mediated photothermal therapy in rat arthritis models demonstrated improved weight-bearing symmetry (measured by incapacitance testing), increased active range of motion at the affected joint, and normalized gait parameters, including stride length and paw print area. These functional improvements correlated with reductions in synovial inflammatory markers, suggesting that molecular and functional outcomes are mechanistically linked (83, 86). The thermal confinement achieved through nanostructure accumulation allows for functional effects not achievable with surface heating alone, which tends to dissipate before reaching deep nociceptors. Research on thermal effects in physical therapy modalities demonstrates that controlled hyperthermia can improve joint mobility in contracture models, with heat application reducing stiffness and increasing range of motion when combined with therapeutic stretching (86). Nanomaterials may enable more controlled dose delivery, though this remains to be validated in human trials.

#### 4.1.2 Fibrotic tissue remodeling

Fibrosis in tendons, ligaments, and joint capsules contributes to pain and restricted motion. Controlled photothermal heating can soften dense collagen networks and reduce fibroblast contractility. When gold nanoshells or nanorods are targeted to fibrotic tissues, near-infrared irradiation produces site-specific collagen denaturation at subablative temperatures, enhancing extensibility and restoring range of motion. Preclinical studies have shown improvements in joint mobility following targeted photothermal intervention, surpassing outcomes observed with conventional physical therapy alone. The mechanistic basis includes thermal modulation of myofibroblast activity and partial disruption of disorganized collagen bundles, followed by more organized remodeling (31).

#### 4.1.3 Wound healing and tissue regeneration

Photothermal stimulation using graphene oxide-hydrogel composites and gold nanoparticle-based systems accelerates wound closure by promoting angiogenesis, collagen deposition, and antimicrobial activity. In diabetic wound models, nanomaterial-mediated photothermal therapy enhanced granulation tissue formation and reduced bacterial burden, thereby accelerating healing compared with controls (81). Mild hyperthermia activates heat shock proteins, improving extracellular matrix assembly and supporting regenerative processes (71).

### 4.2 Ultrasound- and sonodynamic-enhanced musculoskeletal healing

Therapeutic ultrasound has long been used to promote tissue repair, but clinical results have been mixed, particularly in fracture and tendon healing (5). The clinical efficacy of conventional LIPUS for fracture healing remains contested. While early meta-analyses suggested benefit, subsequent large randomized trials (e.g., TRUST trial) found no significant effect on tibial shaft fracture healing time compared to sham treatment. This controversy highlights the importance of critically evaluating nano-enhanced ultrasound approaches. Nanomaterial integration may address limitations of conventional LIPUS by: (1) lowering cavitation thresholds to enable therapeutic effects at lower acoustic intensities; (2) providing localized drug or growth factor release triggered by ultrasound; and (3) enabling real-time imaging feedback to confirm target engagement. However, whether these mechanistic advantages translate into superior clinical outcomes remains to be demonstrated by dedicated randomized trials that have not yet been conducted for nano-enabled ultrasound rehabilitation. Nanomaterials significantly expand the mechanistic repertoire of ultrasound by enabling cavitation enhancement, acoustic droplet vaporization, and ultrasound-triggered drug delivery.

#### 4.2.1 Fracture and tendon healing

Low-intensity pulsed ultrasound (LIPUS) accelerates fracture repair by stimulating chondrocyte proliferation, endochondral ossification, and angiogenesis, though meta-analyses have yielded variable conclusions (5). When combined with nanomaterials, ultrasound effects can be substantially amplified. Nanobubbles and nanodroplets lower the cavitation threshold and increase mechanotransduction signals within the fracture site. Animal studies demonstrate faster healing when ultrasound is combined with nanobubble-enhanced mechanical stimulation and the controlled release of osteoinductive agents, such as BMP-2 (76, 84). Collagen scaffolds embedded with gold nanoparticles have shown enhanced tendon healing when exposed to ultrasound, with improved cellular alignment, increased type I collagen deposition, and significant increases in tensile strength (79, 87).

#### 4.2.2 Cartilage regeneration

Cartilage defects are challenging to treat due to limited intrinsic healing capacity. Microbubble-assisted ultrasound has been used to deliver growth factors, such as TGF-*β*3 and BMP-7, via bubble collapse and cavitation-driven permeabilization. In porcine and caprine models, this approach has restored hyaline-like cartilage with improved glycosaminoglycan content and mechanical properties approaching those of native tissue (30, 76). Such results surpass outcomes typically observed with LIPUS alone.

#### 4.2.3 Ultrasound-Triggered drug delivery

Ultrasound-responsive liposomes have been used to deliver dexamethasone or other anti-inflammatory agents directly into inflamed joints. Research on liposomal interventions for osteoarthritis demonstrates that liposome-mediated delivery systems can improve drug retention and therapeutic efficacy in joint tissues, with liposomes serving as both pharmaceutical carriers and lubricating agents in combinatorial approaches for OA management (88). Ultrasound can also transiently disrupt the blood-brain barrier when paired with microbubbles, enabling delivery of analgesics for neuropathic pain (89).

#### 4.2.4 Nerve regeneration

Ultrasound-stimulated conductive nanofiber conduits have improved Schwann cell proliferation and axonal regrowth in peripheral nerve injury models. Novel aligned piezoelectric nanofibers derived hydrogel nerve guidance conduits with ultrasound-triggered electrical stimulation and controllable drug release have demonstrated acceleration of functional recovery and nerve axonal regeneration in rat models with extended sciatic nerve defects (30). Sonodynamic therapy using porphyrin-loaded carriers has been explored for modulating neuroma tissue in preclinical models, with studies demonstrating the potential to reduce pain behaviors through controlled oxidative signaling (77).

### 4.3 Magnetic nanomaterial-enabled therapy

Magnetic approaches offer strong translational potential because magnetic fields penetrate deep tissues with negligible attenuation, enabling therapeutic action where light and ultrasound are restricted.

#### 4.3.1 Magnetothermal pain modulation and tissue regeneration

Intra-articular SPION injections followed by alternating magnetic field (AMF) exposure can produce controlled hyperthermia within inflamed synovial tissue. Preclinical rabbit and rat models of osteoarthritis have shown reductions in inflammatory markers and improved mobility following magnetothermal therapy (79). Targeted hyperthermia selectively targets hyperproliferative synoviocytes and inflammatory cells while preserving cartilage. AMF-stimulated SPION hydrogels have been shown to enhance chondrogenic differentiation of mesenchymal stem cells by activating mechanosensitive pathways (79). In tendon repair studies, aligned fiber scaffolds embedded with SPIONs and subjected to rotating magnetic fields demonstrated improved tensile properties compared with unstimulated scaffolds (28).

#### 4.3.2 Magnetoelectric neuromodulation

Magnetoelectric nanoparticles have shown efficacy in activating neuronal populations with low-frequency magnetic fields. In rodent models, these particles generated sufficient localized electric potentials to trigger action potentials and preserve muscle mass during denervation (70, 80). These approaches are promising for pain modulation and post-injury neuromuscular rehabilitation. SPION-based hyperthermia has already been approved in Europe for the treatment of glioblastoma (25), demonstrating the clinical feasibility of magnetic nanotherapies.

### 4.4 Nanomaterial-Enhanced electrotherapy and neuromodulation

CNT and PEDOT:PSS coatings dramatically reduce electrode impedance and increase charge-injection capacity, enabling safer, more selective neural stimulation (26, 27). In preclinical models, these coatings substantially reduced the required stimulation voltages, thereby lowering off-target activation and tissue irritation. Injectable conductive hydrogels incorporating graphene oxide or CNTs have accelerated muscle activation and improved responsiveness to electrical stimulation by lowering impedance and improving current distribution (81). Functional outcomes include enhanced muscle force generation and reduced fatigue during repeated stimulation. Conductive nanoparticle-loaded hydrogels implanted into spinal cord lesions enhanced axonal sprouting and improved locomotor recovery in rodent models. When combined with epidural electrical stimulation, these materials amplified functional improvements compared with stimulation alone (90).

### 4.5 Mechanotherapy and nanostructured scaffolds

Aligned electrospun nanofibers direct tenocyte alignment and increase tensile modulus under controlled loading. Mechanically conditioned scaffolds have achieved tensile moduli greater than 150 MPa *in vitro* and superior repair in large-animal models (28, 29). Piezoelectric PVDF nanofibers generate local electric fields under mechanical load, enhancing chondrogenesis and proteoglycan deposition (82). Hybrid scaffolds combining aligned nanofibers and hydrogels have replicated zonal cartilage architecture and improved functional outcomes. Nanofiber scaffolds incorporating hydroxyapatite or bioactive glass nanoparticles improve osteogenesis under dynamic compression (91). In volumetric muscle loss models, aligned nanofibers combined with CNTs and mechanical loading restored substantial contractile function (85).

## 5 Safety, regulatory, ethical, and translational considerations

The integration of nanomaterials into physical rehabilitation raises important questions regarding safety, biocompatibility, regulatory classification, long-term monitoring, and equitable access. Because nanomaterials possess physicochemical characteristics not shared by bulk materials or conventional therapeutics, their interactions with biological systems and the environment must be carefully evaluated. Although many studies support the safety of specific nanomaterials, long-term and repeated-exposure data relevant to rehabilitation remain limited. Translating nano-enabled therapies into clinical practice, therefore, requires a robust understanding of potential risks, regulatory expectations, and ethical implications.

### 5.1 Safety and biocompatibility

Table 3 summarizes the key safety risks and mitigation strategies for nano-enabled rehabilitation therapies.

**Table 3:** Summary of Key safety risks and mitigation strategies for nano-enabled rehabilitation therapies.

Risk Category	Underlying Cause	Clinical Concern	Mitigation Strategy	Evidence
Chronic organ retention	Poorly degradable metals	Long-term RES accumulation	Use biodegradable polymers/MOFs	(92, 93)
Complement activation	Surface charge, PEGylation	Acute infusion reactions	Zwitterionic coatings	(63, 94)
Assay interference	Optical/electrical artifacts	False cytotoxicity readings	Orthogonal assays	(34)
Repeated exposure	Multi-session therapy	Cumulative dose risk	Long-term monitoring; PBPK modeling	(36, 95)

#### 5.1.1 Acute and chronic toxicity

Acute toxicity of nanomaterials depends on size, composition, surface charge, and dose. *In vitro* assays such as MTT, LDH release, and live-dead staining are widely used but can yield misleading results due to nanoparticle interference with assay chemistries (34). Gold nanoparticles, for instance, absorb light in wavelengths used for viability assays, potentially inflating cytotoxicity readings. Complementary assays and strict controls are therefore necessary. Chronic toxicity poses greater concerns for nondegradable or slowly degradable nanomaterials. Gold nanoparticles and certain quantum dots can persist in organs for months to years, particularly within the liver and spleen, where they accumulate in macrophages (35, 92). Although gold exhibits relatively low intrinsic toxicity, the potential for chronic macrophage loading or subtle immune modulation remains incompletely understood (96). Rehabilitation protocols typically involve repeated treatment sessions over weeks to months—a dosing paradigm distinct from oncology applications where single high-dose exposures predominate. A patient receiving twice-weekly magnetothermal therapy for knee osteoarthritis over 12 weeks would receive 24 separate nanoparticle exposures, raising cumulative dose concerns not captured by single-administration toxicity studies. Limited data address this scenario: one preclinical study examined repeated SPION administration (10 doses over 5 weeks) in rats and found progressive hepatic iron accumulation without overt toxicity, but the long-term functional consequences remain unstudied. We recommend that future toxicology assessments incorporate rehabilitation-relevant multi-dose protocols with functional endpoints, including organ function biomarkers measured at intervals throughout the treatment course. Conversely, materials such as PLGA or chitosan degrade into metabolites with established physiological clearance pathways (41), making them more suitable for long-term or repeated rehabilitation interventions.

#### 5.1.2 Organ-Specific effects and clearance

The reticuloendothelial system (RES), particularly Kupffer cells in the liver and macrophages in the spleen, is responsible for clearing most nanoparticles larger than approximately 10 nm (57). Repeated administration can therefore lead to cumulative loading. Renal clearance predominates for ultrasmall nanoparticles (<5–6 nm), although few therapeutic nanomaterials fall within this size range (55). Cardiovascular effects can occur when surface charge promotes platelet activation or when nanoparticles interact with coagulation pathways (97). Nervous system toxicity is generally low unless nanoparticles cross the blood-brain barrier due to pathology or deliberate disruption using ultrasound (89), in which case neuroinflammation must be monitored (32). Nanoparticle biodistribution is strongly influenced by size, shape, and surface chemistry. Biodegradable materials such as PLGA, mesoporous silica, and metal-organic frameworks can degrade over weeks to months, facilitating clearance (93, 98). Nondegradable metallic nanoparticles require careful dose tracking and long-term monitoring.

#### 5.1.3 Immunogenicity and complement activation

Nanoparticles may activate innate immune pathways via pattern recognition receptors or the complement system. Complement activation-related pseudoallergy (CARPA) has been well documented for some liposomal and polymeric formulations and may lead to cardiovascular or respiratory symptoms (94). Repeated dosing of PEGylated nanoparticles can induce anti-PEG antibodies, accelerating clearance and reducing therapeutic efficacy (62, 64). Zwitterionic coatings such as phosphorylcholine can mitigate these effects by reducing protein adsorption (63).

### 5.2 Regulatory frameworks and approval pathways

Table 4 presents the regulatory classification and requirements for nano-enabled physical rehabilitation therapies.

**Table 4:** Regulatory classification and requirements for nano-enabled physical rehabilitation therapies.

Therapy Type	Likely FDA/EMA Classification	Required Evaluations	Key References
Photothermal nanotherapy	Combination product	CMC, ISO-10993, heat-dose mapping	(36, 37)
Magnetothermal therapy	Combination product	Field-dose mapping, biodistribution	(78)
Nano-enhanced ultrasound	Device with nano-excipients	Cavitation threshold mapping	(7)
Conductive nano-electrotherapy	Device with nanocoatings	Electrode safety, stability	(26)
Nanofiber mechanotherapy	Biologic-device hybrid	Scaffold degradation, fatigue	(28)

Nanomaterial-enabled therapies intersect drug, device, and biologic regulatory categories. In the United States, the Center for Drug Evaluation and Research (CDER) oversees nanomaterial-containing pharmaceuticals, while the Center for Devices and Radiological Health (CDRH) regulates medical devices. Many nano-enabled rehabilitation technologies—such as SPIONs used with external magnetic field generators or nanoparticles used with ultrasound—qualify as combination products requiring coordinated review (36). For rehabilitation applications, regulatory classification depends on the primary mode of action. A nano-enhanced TENS unit with CNT-coated electrodes would likely be classified as a Class II medical device requiring 510(k) clearance if substantially equivalent to predicate devices. In contrast, injectable SPION formulations for magnetothermal therapy would require drug/device combination product review, potentially under FDA's Office of Combination Products. Notably, outpatient rehabilitation settings present unique considerations: repeated patient self-administration, variable treatment compliance, and limited clinical monitoring may affect risk-benefit assessments. The existing regulatory guidance does not specifically address nano-enabled rehabilitation devices, representing a gap that may delay clinical translation. Both FDA and EMA emphasize comprehensive physicochemical characterization, strict manufacturing controls, and detailed risk-benefit assessments for nanomaterials (36, 37). Key requirements include measurement of size distribution, surface charge, aggregation state, chemical composition, impurities, and stability under physiological conditions. For therapies involving repeat administration—such as photothermal or magnetothermal interventions delivered over multiple rehabilitation sessions—regulators may require extended follow-up periods of 2–5 years to monitor chronic organ accumulation or subtle immune perturbations (36, 94). Physiologically based pharmacokinetic (PBPK) modeling is increasingly used to predict distribution and clearance across patient populations (95).

### 5.3 Ethical considerations

Nanomedicine presents conceptual challenges for patients trying to understand the nature of nanoscale interventions. Because public familiarity with nanoparticles is limited, informed consent must clearly describe nanoparticle behavior, distribution, possible organ retention, and uncertainties regarding long-term effects (99). Patients require accessible explanations that avoid overstating benefits or downplaying unknowns. Advanced nanotechnologies may increase the cost of rehabilitation interventions if sophisticated equipment or proprietary nanomaterials are required (100). Without careful policy planning, these therapies risk exacerbating disparities between well-resourced and underserved clinical settings. Conversely, if nanomaterials enable more efficient rehabilitation with fewer clinic visits, they could reduce total healthcare burdens. Manufacturing and disposal of nanomaterials raise environmental concerns, particularly regarding inhalation exposure during production or release into wastewater systems (101). Adoption of green chemistry approaches and biodegradable materials can reduce these risks. Some nano-enabled modalities, particularly those involving neural modulation or performance augmentation, challenge the ethical boundary between therapeutic intervention and enhancement (102).

### 5.4 Translational challenges and future directions

The translational roadmap for nano-enabled rehabilitation technologies involves a stepwise pathway from nanomaterial synthesis through mechanistic validation, preclinical rehabilitation models, clinical early-phase trials, scalable manufacturing, and long-term post-market surveillance. Nanomaterial synthesis often involves conditions that are difficult to scale while maintaining uniformity. Minor variations in temperature, pH, reagent purity, or mixing rates can alter size distribution, surface coatings, or functionalization efficiency (59). Scalable techniques such as microfluidics may improve reproducibility. Rehabilitation applications require precise control of external energy fields—light fluence, ultrasound frequency and intensity, magnetic field amplitude, and electrical current density. Variability across studies makes comparison difficult and hampers meta-analytic assessment (103). Standardized reporting frameworks are necessary for clinical translation. Individual differences in tissue composition, inflammation, vascularization, and immune function influence nanoparticle distribution and responsiveness to external stimuli (57). Personalized dosing—potentially guided by digital imaging, biosensors, or computational modeling—will likely be required. Next-generation designs aim to integrate sensing and actuation within a single platform—such as nanoparticles that release drugs in response to mechanical loading or inflammation (104). Machine learning can identify nanoparticle design parameters that optimize therapeutic properties (105). Digital twins may simulate individual patient responses to nano-enabled physical therapies (106), guiding personalized protocols (107).

## 6 Conclusions

Nanomaterial-enabled physical rehabilitation represents a convergence of materials science, biophysics, and clinical practice, offering new ways to modulate biological processes central to tissue repair, neuromuscular recovery, and pain management. Engineered nanomaterials can localize and amplify externally applied stimuli such as light, ultrasound, magnetic fields, electrical currents, and mechanical forces, enabling biological effects not achievable through conventional modalities alone. Preclinical evidence supports the potential of nanomaterial-enabled photothermal therapy, sonodynamic and ultrasound-enhanced repair, magnetic hyperthermia and mechanostimulation, nano-enhanced electrotherapy, and nanostructured scaffolds for mechanotherapy and regeneration.

However, significant challenges remain. Safety considerations—including chronic organ retention, immune activation, and repeated-exposure risks—must be addressed through rigorous long-term studies. Regulatory pathways for combination products require coordinated assessment of device and nanomaterial components. Ethical issues, including equitable access and enhancement concerns, merit ongoing attention. Manufacturing reproducibility, dose standardization, and personalization strategies must be developed to ensure predictable therapeutic outcomes. By integrating mechanistic understanding with empirical evidence and responsible translational strategies, nanomaterial-enabled rehabilitation, while currently at the preclinical and early translational stage, demonstrates proof-of-concept potential to complement existing physical therapy approaches. Realizing the vision of precise, adaptive, and molecularly informed rehabilitation interventions will require rigorous clinical validation, standardized protocols, and resolution of the safety and manufacturing challenges outlined in this review.
